# Low-Salt Diet Attenuates B-Cell- and Myeloid-Cell-Driven Experimental Arthritides by Affecting Innate as Well as Adaptive Immune Mechanisms

**DOI:** 10.3389/fimmu.2021.765741

**Published:** 2021-12-03

**Authors:** Bettina Sehnert, Sandy Pohle, Cornelia Heuberger, Rita Rzepka, Maximilian Seidl, Falk Nimmerjahn, Nina Chevalier, Jens Titze, Reinhard E. Voll

**Affiliations:** ^1^Department of Rheumatology and Clinical Immunology, Medical Center–University of Freiburg, Faculty of Medicine, University of Freiburg, Freiburg, Germany; ^2^Department of Medicine 3, Friedrich-Alexander-University of Erlangen-Nuremberg, Universitätsklinikum Erlangen, Erlangen, Germany; ^3^Institute for Surgical Pathology, Department of Pathology, Medical Center-University of Freiburg, Faculty of Medicine, University of Freiburg, Freiburg, Germany; ^4^Institute of Pathology, Heinrich-Heine University and University Hospital of Düsseldorf, Düsseldorf, Germany; ^5^Institute of Genetics, Department of Biology, University of Erlangen-Nuremberg, Erlangen, Germany; ^6^Interdisciplinary Center for Clinical Research and Department of Nephrology and Hypertension, Nikolaus-Fiebiger Center for Molecular Medicine, Friedrich-Alexander-University, Erlangen, Germany; ^7^Cardiovascular and Metabolic Disorders, Duke-NUS Medical School, Singapore, Singapore; ^8^Center for Chronic Immunodeficiency (CCI) Freiburg, Medical Center-University of Freiburg, Faculty of Medicine, University of Freiburg, Freiburg, Germany

**Keywords:** collagen-induced arthritis, serum transfer-induced arthritis, diet, sodium chloride, cytokines

## Abstract

A link between high sodium chloride (salt) intake and the development of autoimmune diseases was previously reported. These earlier studies demonstrated exacerbation of experimental autoimmune encephalomyelitis and colitis by excess salt intake associated with Th17- and macrophage-mediated mechanisms. Little is known about the impact of dietary salt intake on experimental arthritides. Here, we investigated if salt restriction can exert beneficial effects on collagen-induced arthritis (CIA) and K/BxN serum transfer-induced arthritis (STIA). CIA depends on both adaptive and innate immunity, while STIA predominantly mimics the innate immune cell-driven effector phase of arthritis. In both models, low salt (LS) diet significantly decreased arthritis severity compared to regular salt (RS) and high salt (HS) diet. We did not observe an aggravation of arthritis with HS diet compared to RS diet. Remarkably, in STIA, LS diet was as effective as IL-1 receptor blocking treatment. Complement-fixing anti-CII IgG2a antibodies are associated with inflammatory cell infiltration and cartilage destruction. LS diet reduced anti-CII IgG2a levels in CIA and decreased the anti-CII IgG2a/IgG1 ratios pointing toward a more Th2-like response. Significantly less inflammatory joint infiltrates and cartilage breakdown associated with reduced protein concentrations of IL-1 beta (CIA and STIA), IL-17 (CIA), and the monocyte chemoattractant protein-1 (MCP-1) (CIA) were detected in mice receiving LS diet compared to HS diet. However, we did not find a reduced IL-17A expression in CD4^+^ T cells upon salt restriction in CIA. Analysis of mRNA transcripts and immunoblots revealed a link between LS diet and inhibition of the p38 MAPK (mitogen-activated protein kinase)/NFAT5 (nuclear factor of activated T-cells 5) signaling axis in STIA. Further experiments indicated a decreased leukodiapedesis under LS conditions. In conclusion, dietary salt restriction ameliorates CIA and STIA, indicating a beneficial role of LS diet during both the immunization and effector phase of immune-mediated arthritides by predominantly modulating the humoral immunity and the activation status of myeloid lineage cells. Hence, salt restriction might represent a supportive dietary intervention not only to reduce cardiovascular risk, but also to improve human inflammatory joint diseases like rheumatoid arthritis.

## Introduction

Rheumatoid arthritis (RA) is a chronic autoimmune disease in which complex interactions of adaptive and innate immune cells cause chronic joint inflammation resulting in a destruction of cartilage and bone (1). Genetic and environmental factors such as smoking, infections, and dietary components affect RA manifestation and severity (1–4). RA is linked to a higher risk for cardiovascular diseases (CVD) accompanied with increased mortality (5, 6).

The “Western diet”, containing high amounts of sodium chloride (NaCl, referred to as salt), represents an important risk factor in inflammatory autoimmune diseases (7). A link between excess salt intake and exacerbated disease in murine experimental autoimmune encephalomyelitis (EAE) (8, 9) and colitis models was reported (8, 10, 11). High-salt diet aggravates EAE most likely due to an increased response of T helper 17 cells (Th17), which is induced by the p38/MAPK pathway involving the tonicity enhancer binding protein (TonEBP or nuclear factor of activated T-cells 5 [NFAT5]) and the salt-sensing serum glucocorticoid kinase 1 (SGK1) (9). In addition, salt-sensitive innate immune cells such as macrophages critically contribute to increased inflammation in EAE (8).

While detrimental in (auto-) inflammatory diseases, increased interstitial salt concentrations at sites of skin infections boost the bactericidal response in macrophages by an NFAT5-dependent mechanism and thereby promote host defense (12). In particular, macrophages may act as local sensors and physiological regulators of electrolyte composition in the interstitium of the skin (13, 14).

Little information exists on the impact of salt intake on RA. In a nested case–control study, risk for RA development was found to be doubled in smokers consuming highly salted food (15). Furthermore, it was discussed that restricted salt intake may dampen inflammatory responses in RA and SLE patients by regulating adaptive immunity (16). So far, comprehensive studies on the clinical, histological, and immunologic effects of salt intake in arthritides are rare. Moderate salt intake did not aggravate clinical manifestation using the T-cell-dependent model of collagen-induced arthritis (CIA) when compared to regular salt intake (11) while CIA mice exposed to excess salt consumption showed exacerbated arthritis *via* Th17 polarization (17).

Studies investigating the effects of restricted salt intake on experimental arthritides are missing. In this investigation, we were particularly interested in the question if and how a low salt (LS) diet can improve the clinical disease course and prevent joint destruction of CIA and K/BxN serum transfer-induced arthritis (STIA) compared to a regular salt (RS) and a high salt (HS) diet. CIA is induced by immunization with bovine collagen type II (CII) and characterized by an initial phase dominated by humoral/cell-mediated adaptive immunity and autoantibody production followed by an innate effector phase, whereas in STIA, the latter plays the dominant role (18, 19). In addition, we investigated the relevance of salt restriction on cell migration in the model of thioglycollate-induced peritonitis. Furthermore, the impact of NaCl restriction on TNF-alpha and IL-6 expression in lipopolysaccharide (LPS)-stimulated murine monocytes and IL-1 beta-induced E-selectin expression in an endothelial cell line was investigated *in vitro*.

## Materials and Methods

### Animals

C57BL/6J and DBA/1J were purchased from Janvier Laboratories, Le Genest*-*Saint-Isle, France. Mice were maintained under conventional housing with five mice per cage and were used between 8 and 12 weeks of age at the time of the experiments. Mice were maintained under controlled 12-h light/12-h dark cycles. The respective diets and the drinking water were inspected regularly and changed twice per week. Blood collection and mouse handling were reduced to a minimum to avoid stress.

### Salt Diets in Arthritis Models

Mice were fed with rodent chow containing different concentrations of NaCl. Group 1 received a HS diet using HS rodent chow (4% NaCl, ssniff^®^ EF R/M High Sodium, Ssniff, Soest, Germany) and tap water supplemented with 1% NaCl (HS diet). Group 2 received a LS diet: LS chow (<0.1% NaCl, Ssniff^®^ EF R/M Sodium deficient) with tap water (LS diet) and Group 3 was fed with regular chow (0.5% NaCl, Kliba Nafag, Kaiseraugst, Switzerland) with tap water (RS diet). Respective diets were started 2 weeks before arthritis induction and were continued for 47 days in CIA and for 8 days in STIA. CIA was induced as previously described (20). K/BxN serum was used to induce joint inflammation in STIA (21). In STIA, a group of HS-treated animals received intraperitoneal injections of 100 mg/kg anakinra (Kineret^®^, Swedish Orphan Biovitrum GmbH, Martinsried, Germany) simultaneously with arthritis induction as well as on days 1, 2, and 3.

### Scoring of Arthritic Paws

Arthritis was macroscopically scored by two independent investigators in a blinded fashion. Arthritis score was graded on a scale of 0–4 for each paw: 0 = no swelling; 1 = swelling of one joint; 2 = moderate swelling of > one joint; 3 = extensive swelling > one joint; 4 = severe swelling of the entire paw. Each paw was individually graded and scores were added up for an overall arthritis score between 0 and 16 per mouse. Mean arthritis score is expressed as mean of scoring points per group. The area under the curve (AUC) of the arthritis score was calculated from day 18 to day 47 (CIA) and day 0 to day 8 (STIA).

### Preparation of Paws

At sacrifice day, mice were euthanized using CO_2_. Hind paws were dissected, fixed in 4% paraformaldehyde (PFA) for 24 h, and decalcified in 10% EDTA and 100 mM Tris, pH 7.5, for 2 weeks under constant shaking (22). Decalcified paws were stored in 70% ethanol until further processing. Front paws were dissected, snap frozen in liquid N_2_, and stored at −80°C until further processing.

### Preparation of Paw Tissue Extracts

Extracts of front paws were prepared using a tissue lyserLT (Qiagen, Hilden, Germany) according to the manufacturer’s recommendations. In brief, dissected front paws were homogenized in 1 ml of homogenization buffer [PBS with 1 mM PMSF, 1× Protease Inhibitor EDTA-free tablets (Sigma, Taufkirchen, Germany)] per 200 mg paw weight, with stainless steel beads (5 mm) for 15 min at 50 Hz. The homogenates were centrifuged twice at 13,000×*g* for 10 min at 4°C and the supernatants were used for cytokine analysis.

### Cytokine ELISA

Concentrations of IL-1β, IL-6, IL-17A, IL-10, RANTES [Regulated and Normal T cell Expressed and Secreted or CCL5 (C-C Motif Chemokine Ligand 5)], and MCP-1 (monocyte chemoattractant protein-1) were analyzed in joint tissue extracts of front paws by ELISA DuoSets (R&D Systems, Wiesbaden, Germany) according to the manufacturer’s instructions. The total protein concentrations were quantified by BCA assay (Thermo Fisher Scientific, Schwerte, Germany).

### Histological Assessment of Joints

Fixed and decalcified hind paws were embedded in paraffin; 2-µm-thick sections were prepared and placed on SuperFrost Plus slides (Thermo Fisher Scientific, Braunschweig, Germany) (22). The sections were stained with hematoxylin/eosin (HE) (Sigma, Taufkirchen, Germany) or alcian blue (Sigma, Taufkirchen, Germany). The sections were scored for inflammation (grade 0–3): 0, no changes; 1, mild hyperplasia and infiltration; 2, moderate infiltration; 3, extensive infiltration, and for cartilage damage (grade 0–3): 0, no destruction; 1, mild destruction; 2, moderate destruction; 3, extensive destruction by two independent investigators in a blinded fashion. Scores for individual criteria were added up for an overall joint inflammation score between 0 and 6 per mouse. The data were expressed as mean histological score. Joint images were acquired with an ApoTome microscope Zeiss (Zeiss, Oberkochen, Germany) using the Zeiss software Zen 2012.

### Immunohistochemical Stainings

Immunohistochemical (IHC) staining for IL-17A (# 91649, Abcam, Cambridge, UK), IL-1β (# 9722 Abcam, Cambridge, UK), and F4/80 (# 100790, Abcam, Cambridge, UK) was performed using the peroxidase-based EnVision+ System-HRP (DAB) Kit (Kit K4010, Dako, Hamburg, Germany). Briefly, the joint sections were deparaffinized, rehydrated, and subsequently subjected to an antigen retrieval according to the manufacturer’s instructions. To block endogenous peroxidases, 3% H_2_O_2_ in 60% methanol were placed on the sections. Non-specific binding of the antibodies was reduced by blocking with 3% BSA in PBS/0.5% Tween20. The primary antibodies or rabbit polyclonal IgG for isotype control (# 27472, Abcam, Cambridge, UK) were applied. To detect IL-17A or IL-1β, 5 µg/ml of anti-mouse IL-17A antibody or anti-mouse IL-1β, respectively, diluted in blocking buffer were used. The anti-mouse F4/80 was diluted 1/100 in blocking buffer. All sections were incubated overnight at 4°C. Specimens were incubated with Polymer-HRP labeled anti-rabbit (Kit K4010, Dako, Hamburg, Germany) for 30 min at room temperature. Then, 3,3′-diaminobenzidine 4-HCl (DAB) solution was applied on the specimen and slides were incubated for 7 min. Sections were counterstained with Meyer’s Hematoxylin. At the end, the specimens were mounted using an aqueous-based mounting medium and a cover slip. Images were acquired with an ApoTome microscope Zeiss (Zeiss, Oberkochen, Germany) using the Zeiss software Zen 2012. The histological sections were assessed by semi-quantitative analysis (23). Sections of four randomly chosen mice of each group from one experiment were used and three fields per section were analyzed. The area of DAB-positive cells was analyzed by loading images into ImageJ (v.1.47). By setting the threshold to appropriate values, DAB-positive cells were displayed. The values were expressed as percentage of DAB-positive areas (highlighted area/non-highlighted area × 100).

### Blood Collection

Blood of mice was collected in serum vacutainer tubes (BD, Heidelberg, Germany), centrifuged for 10 min at 13,000 × *g*, and stored at −20°C.

### Measurement of Anti-CII IgG, IgG1, and IgG2a

Serum levels of anti-CII IgG, anti-CII IgG2a, and anti-CII IgG1 were measured by ELISA. Microtiter plates were coated with 10 μg/ml native bovine CII, blocked with 2% BSA/PBS, and then incubated with diluted mouse sera. A serum dilution of 1:1,000 in PBS was assayed for IgG and IgG1, as well as a 1:4,000 dilution for IgG2a. Bound IgG and IgG subclasses were detected by incubation with HRPO-conjugated rabbit anti-mouse IgG, IgG1, and IgG2a, respectively. The HRPO-labeled antibodies were diluted 1:4,000 in 1% BSA/PBS. For color development, ABTS substrate was added, and the optical density (OD) was read at 405 nm with an ELISA Reader Infinite F50 (Tecan, Crailsheim, Germany). A standard serum was generated using a pool of sera containing high-titer anti-CII antibodies and considered equivalent to 100 arbitrary units/ml. A standard curve was plotted using dilutions of 1:500 (0.2 units/ml), 1:1,000 (0.1 units/ml), 1:1,250 (0.08 units/ml), 1:2,000 (0.05 units/ml), and 1:4,000 (0.025 units/ml). The Michaelis–Menten equation was used to convert optical density values to units.

Goat anti-mouse IgG horseradish peroxidase (HRPO), goat anti-mouse IgG1 HRPO, and goat anti-mouse IgG2a HRPO (Southern Biotech products from Biozol, Eching, Germany) were used as detection antibodies in ELISA.

### *In Vitro* Analysis of TNF-Alpha, IL-6, and E-Selectin Expression

Monocytes were isolated from spleens of C57BL/6J mice and incubated in media with different NaCl concentrations, ranging from 25 mM to 165 mM (24). Osmolality of solutions was determined by an osmometer (Osmomat 030, Gonotec, Berlin, Germany). Equi-osmolalic solutions containing mannitol (ranging from 20 mM to 280 mM) were prepared and used as controls to identify NaCl-dependent effects. After an incubation time of 24 h, monocytes were stimulated by LPS (500 ng/ml) for further 18 h. Supernatants were harvested and secreted mediators were analyzed by cytometric bead assay (LegendPlex Mouse Inflammation Panel, BioLegend Europe BV, Amsterdam, Netherlands). The pro-inflammatory cytokines TNF-alpha and IL-6 were detectable in LPS stimulated mouse monocytes, whereas IL-1 beta and MCP-1 were not readily detectable. Adherent monocytes were detached by Trypsin 0.25%/EDTA 0.02% (Pan-Biotech GmbH, Aidenbach, Germany) according to the manufacturer’s instructions. Afterwards, cells were resuspended in an appropriate volume of pre-warmed medium and stained by 0.4% Trypan blue solution (Thermo Fisher Scientific, Schwerte, Germany) to count viable and dead cells in a hematocytometer (25). E-selectin expression on murine mesenteric lymph node–derived endothelioma cells (*mlEnds*) were analyzed by culturing the cells as described above for 24 h. Afterwards, E-selectin expression was induced by 40 ng/ml IL-1 beta incubation for an additional 4 h. mlENDs were stained with an anti-CD62E antibody (BioLegend Europe BV, Amsterdam, Netherlands). Propidium iodide (PI) (Thermo Fisher Scientific, Schwerte, Germany) staining (0.5 µg/sample) was performed to assess cell viability by flow cytometry (26). The samples were analyzed by a Coulter Gallios flow cytometer (BeckmanCoulter, Krefeld, Germany). E-selectin was quantified as mean fluorescence intensity (MFI).

The impact of IL-1 beta-induced activation of nuclear factor-kappaB (NF-kappaB), p38, and c-Jun-NH2-terminal kinase (JNK) on E-selectin expression was examined in *mlEND. mlENDs* were cultured at 37°C and 7.5% CO_2_ in DMEM medium supplemented with 10% FCS, 1% penicillin/streptomycin, 4 mM L-glutamine, and 1 mM sodium pyruvate (all cell culture reagents were supplied by Thermo Fisher Scientific, Schwerte, Germany) and pre-treated with IKK-16 (500 nM) (# S2882, Biozol, Eching, Germany), SB203580 (10 µM) (#5633s, Cell Signaling, Frankfurt a.M., Germany), and SP6000125 (10 µM) (#8177, Cell Signaling) or DMSO as control for 1 h followed by incubation with IL-1 beta (40 ng/ml). E-selectin expression was analyzed as described above.

### Statistical Analysis

Statistical analysis was performed using the GraphPad Prism 9 software. Results are shown as mean ± standard error of the mean (SEM). In the case of normal distribution, unpaired Student’s *t*-test (two groups) and one-way ANOVA (three groups) were used to test one parameter. Repeated measurements were analyzed with two-way ANOVA followed by *post-hoc* analysis. Kaplan–Meier Estimator was used to show differences in incidences of experimental arthritis performing log rank (Mantel–Cox). Statistical significance was defined as *p* ≤ 0.05 and rating of statistical significance was defined as *p ≤ 0.05; **p ≤ 0.01; ***p ≤ 0.005; ****p ≤ 0.0001.

## Results

### NaCl Restriction Decreases TNF-Alpha and IL-6 Levels in Murine Monocytes *In Vitro*

**Figure 1:**
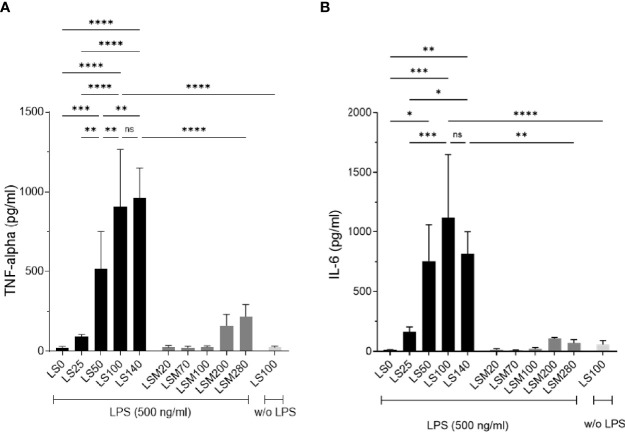
Lowering NaCl concentration in cell culture medium inhibits TNF-alpha and IL-6 production of monocytes. Low salt (LS) medium was prepared as described elsewhere (24) and used as described in **Table 1**. Murine monocytes from spleens of C57BL/6J mice were isolated and cultured in solutions under respective conditions for 24 h before TNF-alpha and IL-6 expression was induced by LPS (500 ng/ml) for 18 h. TNF-alpha **(A)** and IL-6 **(B)** concentrations were analyzed in the supernatants by cytometric-bead assays. *p < 0.05, **p < 0.01, ***p < 0.001, ****p < 0.0001, ns = non-significant. *p*-values were calculated by one-way ANOVA followed by Bonferroni’s multiple comparisons test. All data were expressed as ± SEM from four independent experiments with technical duplicates.

Recent studies showed that increased salt exposure affects not only lymphoid but also myeloid effector functions (8, 11). The cytokines TNF-alpha and IL-6 deriving predominantly from myeloid cells promote not only RA but also experimental arthritides (27–29). First, we investigated the effect of low salt exposure on TNF-alpha and IL-6 expression on murine monocytes *in vitro*. Murine monocytes were pre-incubated for 24 h in medium containing different concentrations of NaCl (24). The NaCl concentrations ranged from 25 mM NaCl (LS0), 50 mM NaCl (LS25), 75 mM NaCl (LS50), 125 mM NaCl (LS100 corresponding to regular salt condition with 299 mOsm/kg) to 165 mM NaCl (LS140). Osmolalities (mOsm/kg) of the solutions are outlined in **Table 1**. Cytokine expression was induced by LPS stimulation and cytokine concentrations were measured after 18 h in the cell culture supernatants. After LPS stimulation, cell viability was analyzed by Trypan blue exclusion assay and revealed an overall viability of ≥93%. There were no significant differences in viability between the LS0, LS100, and LS140 groups (**Supplementary Figure 1A**). TNF-alpha and IL-6 concentrations are presented in **Figures 1A, B**, respectively. As expected, LPS significantly upregulated TNF-alpha and IL-6 expression under LS100 conditions compared to unstimulated monocytes (w/o LPS). Stimulation of monocytes in LS140 medium did not increase TNF-alpha and IL-6 production compared to stimulation in LS100 medium. When comparing LS50 to medium ≥LS100, TNF-alpha expression was significantly downregulated but not IL-6. In contrast, a significant reduction of TNF-alpha (**Figure 1A**) and IL-6 (**Figure 1B**) production was observed in monocytes cultured in LS0 medium compared to monocytes cultured in medium ≥LS50. Also, cells cultured in LS25 medium secreted significantly less cytokines compared to cells cultured in medium containing ≥LS100. To assess the specific effects of NaCl, we also analyzed cytokine expression of cells cultured in solutions containing iso-osmolalic concentrations of mannitol (named as LSM solutions). In comparison to the respective NaCl-containing solutions, mannitol supplementation resulted in overall significantly lower TNF-alpha and IL-6 concentrations indicating the potency of NaCl to induce pro-inflammatory cytokine expression. It is to be noted that monocyte-derived mediators such as IL-1 beta or MCP-1 were not detectable in the cell culture supernatants under the experimental conditions used.

**Table 1:** Compositions of cell culture medium containing different concentrations of NaCl and mannitol.

Composition of solutions			
Low salt (LS) medium	NaCl (mM) added	mOsm/kg	Name
LS	0	98	LS0
LS	25	150	LS25
LS	50	202	LS50
LS	100	299	LS100
LS	140	387	LS140
**Low salt (LS) medium**	**Mannitol (mM) added**	**mOsm/kg**	**Name**
LS	20	112	LSM20
LS	70	165	LSM70
LS	100	198	LSM100
LS	200	289	LSM200
LS	280	361	LSM280

Low salt (LS) medium was prepared as described elsewhere (24), supplemented with respective concentrations of NaCl and used for in vitro assays as follows: LS0 contains 25 mM NaCl (LS25 ≙ 50 mM NaCl, LS50 ≙ 75 mM NaCl, LS100 ≙ 125 mM NaCl, LS140 ≙ 165 mM NaCl). LS100 corresponds to regular salt conditions, LS140 to high salt conditions, and ≤ LS50 to low salt conditions. To compare effects of NaCl solutions to an isosmotic substance, LS medium was supplemented with mannitol to obtain equi-osmolalic solutions. Osmolality (mOsm/kg) of respective solutions was measured (third column).

Taken together, these data reveal that the expression of the myeloid-derived pro-inflammatory cytokines TNF-alpha and IL-6 can be decreased by lowering the NaCl concentration in the surrounding milieu.

### E-Selectin Expression Is Decreased by NaCl Restriction in Activated Mouse Endothelial Cells *In Vitro* in a NF-KappaB Independent Manner

**Figure 2:**
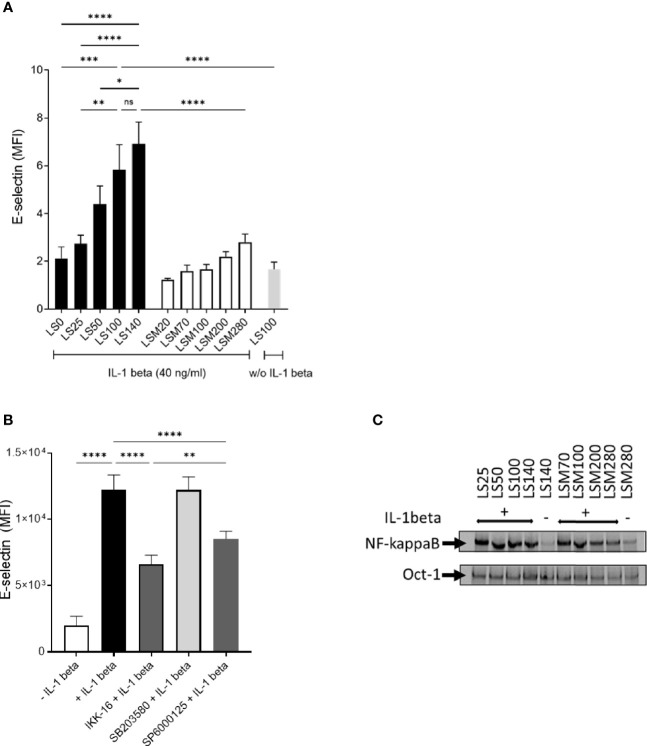
Lowering NaCl concentration in cell culture medium inhibits cytokine-induced E-selectin expression on endothelial cells in a NF-kappaB-independent manner. **(A)** Murine mesenteric lymph node–derived endothelioma cells (*mlEnds*) were cultured in solutions under respective conditions for 24 h before E-selectin was induced by IL-1 beta (40 ng/ml) for 4 h. E-selectin expression on *mlEND* cell surface was analyzed by flow cytometry and expressed as mean fluorescence intensities (MFI). *p < 0.05, **p < 0.01,***p < 0.001, ****p < 0.0001, ns = non-significant. *p*-values were calculated by one-way ANOVA followed by Bonferroni’s multiple comparisons test. All data were expressed as ± SEM from three independent experiments with technical duplicates. **(B)** *mlENDs* were pre-treated with an NF-kappaB inhibitor (IKK16;500 nM), a p38 inhibitor (SB203085;10 µM), and a JNK inhibitor (SP6000125; 10 µM) for 1 h before E-selectin expression was induced by IL-1 beta. MFIs of E-selectin are shown. ****p < 0.0001. *p*-values were calculated by one-way ANOVA followed by Bonferroni’s multiple comparisons test. All data were expressed as ± SEM from three independent experiments with technical duplicates. **(C)** Electrophoretic mobility shift assay (EMSA) to analyze NF-kappaB nuclear DNA-binding activity under NaCl restriction. *mlENDs* were cultured in respective media (LS25, LS50, LS100, LS140, LSM20, LSM70, LSM200, and LSM280) and incubated for 1 h with 40 ng/ml IL-1 beta. Nuclear extracts were applied to NF-kappaB and Oct-1 EMSA. Oct-1 was used as loading control. One representative independent experiment out of four experiments is shown.

Endothelial cells are gate-keepers in the inflammatory immune response and control the extravasation of leukocytes from the periphery into the inflamed tissue. Cytokines such as IL-1 beta and TNF-alpha stimulate the expression of adhesion molecules such as E-selectin mediating leukocyte rolling (30). In order to test if extracellular NaCl can modulate E-selectin expression in endothelial cells, we cultured *mlEND* cells in varying NaCl concentrations (LS0, LS25, LS50, LS100, and LS140) for 24 h before stimulating them with IL-1 beta for an additional 4 h to induce E-selectin expression. NaCl-dependent effects were compared to mannitol-induced iso-osmotic effects. Flow cytometric analyses revealed that IL-1 beta stimulation significantly increased E-selectin expression in *mlEND* cells under LS100 condition compared to unstimulated cells (w/o IL-1 beta) (**Figure 2A**). Overall, we observed a dose-dependent reduction of E-selectin expression when lowering the NaCl concentration in the culture medium. Incubation of *mlEND* cells in low NaCl conditions (≤ LS25) significantly decreased E-selectin expression compared to regular and high NaCl conditions (LS100 and LS140, respectively) (**Figure 2A**). In contrast, high NaCl conditions (LS140) did not lead to a significant increase of E-selectin expression in *mlEND* cells compared to regular NaCl conditions (LS100) (**Figure 2A**). Moreover, incubation of *mlEND* cells with LS100 and LS140 resulted in a significantly higher E-selectin expression than in mannitol-containing solutions LSM200 and LSM280, respectively, indicating a hyperosmolalic effect specifically by NaCl on E-selectin expression (**Figure 2A**). PI staining of *mlENDs* revealed no significant differences of viability under NaCl restriction (**Supplementary Figure 1B**).

Previous studies showed that E-selectin expression is transcriptionally regulated *via* NF-kappaB and JNK pathways (31). Hence, we investigated the relevance of the NF-kappa, p38, and JNK signaling pathways on E-selectin expression in IL-1 beta-stimulated *mlEND* cells by inhibiting these pathways. To this end, *mlENDs* were treated with IKK-16 (500 nM), SB203580 (10 µM), or SP6000125 (10 µM), before stimulation with IL-1 beta in DMEM medium to induce E-selectin expression. Flow cytometric analysis demonstrated significantly reduced E-selectin expression by NF-kappaB and JNK inhibition compared to vehicle-treated cells, whereas p38 inhibition did not result in reduced E-selectin expression on the surface of *mlEND* cells (**Figure 2B**). We next measured the NF-kappaB DNA-binding activity of nuclear extracts from *mlEND* cells cultured in LS25, LS50, LS100, and LS140 media as well as cultured in LSM20, LSM70, LSM100, and LSM280 media by EMSA. Oct-1 DNA-binding activity was used as loading control. IL-1 beta stimulation induced an increase of NF-kappaB DNA-binding activity compared to unstimulated mlENDs cultured in LS140. No alterations of NF-kappaB DNA binding activities were observed between the groups of IL-1 beta-stimulated mlENDs pre-incubated in LS25, LS50, LS100, and LS140 media. These observations were replicated in LSM20-, LSM70-, LSM100-, and LSM280-treated cells (**Figure 2C**). Furthermore, the NF-kappaB DNA binding activities were unchanged by salt restriction in unstimulated mlENDs (data not shown). Altogether, lowering the NaCl concentration in the surrounding milieu of cultured endothelial cells downregulated E-selectin expression, which might contribute to controlling leukocyte extravasation.

### Low-Salt Diet Ameliorates Severity and Incidence of Collagen-Induced Arthritis

**Figure 3:**
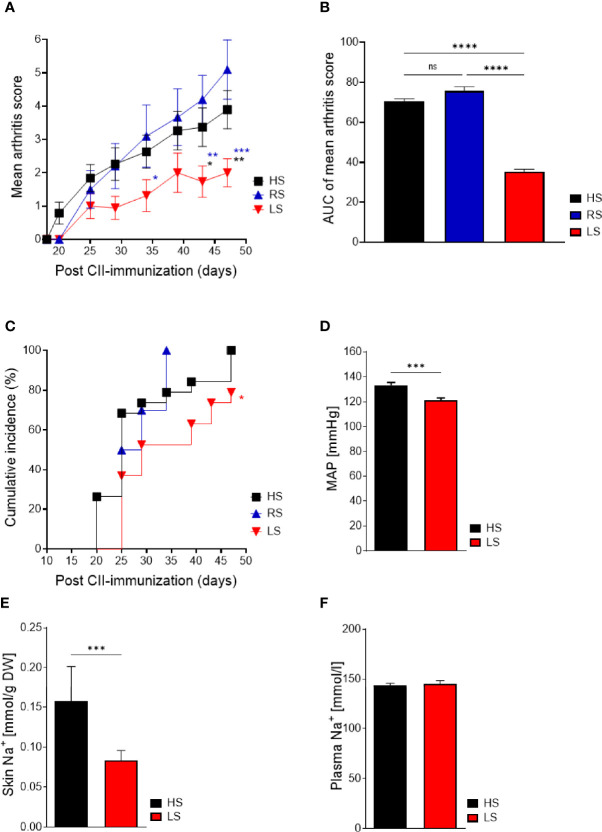
Low-salt diet reduces severity and incidence of arthritis and salt-related effects in collagen-induced arthritis. Mice were exposed to low salt (LS), regular salt (RS), and high salt (HS) diets for 2 weeks before CII immunization and continued for further 47 days. **(A)** Mean arthritis score of CIA mice under respective diets (LS, *n* = 19; RS, *n* = 9; HS, *n* = 19). *p < 0.05, **p < 0.01,***p < 0.001; blue asterisk: LS versus RS, black asterisk: LS versus HS. *p*-values were calculated by two-way ANOVA followed by Bonferroni’s multiple comparisons test. **(B)** Area under curve of mean arthritis score. ****p < 0.0001. *p*-values were calculated by one-way ANOVA followed by Bonferroni’s multiple comparisons test. **(C)** Cumulative incidence of CIA with LS, RS, and HS diet (LS, *n* = 19; RS, *n* = 9; HS, *n* = 19). *p < 0.05, ns = non-significant. Kaplan–Meier method was used to calculate differences in incidences of experimental arthritis performing log rank (Mantel-Cox). **(D)** Blood pressure measurements of CIA mice kept under LS and HS diets. ***p < 0.001. **(E)** Levels of Na^+^ (mmol/gr dry weight) in skin of CIA mice with LS and HS diets. ***p < 0.001**. (F)** Plasma Na^+^ levels (mmol/l). *p*-values were calculated by unpaired Student’s *t*-test **(D–F)**. All data were expressed as ± SEM.

In susceptible mouse strains, immunization with a CII/CFA emulsion results in an immune response against CII followed by joint inflammation (32). To examine the effects of NaCl on inflammatory diseases *in vivo*, we investigated the effects of a reduced nutritional salt intake on the clinical manifestations of CIA compared to a regular or a high-salt diet. DBA/1 mice were fed with different salt diets 2 weeks before CII immunization and for an additional 47 days. In CIA, LS diet significantly reduced clinical severity compared to RS and HS diets (**Figure 3A**). In contrast to the EAE studies described by others (8, 9), HS diet did not aggravate disease course compared to RS diet in CIA (**Figure 3A**). The significant treatment effect of a reduced NaCl intake is further shown by the reduced area under the curve (AUC) of the arthritis score (**Figure 3B**). A log rank (Mantel-Cox) test revealed that CII-immunized mice exposed to a salty diet developed arthritis with a higher incidence rate compared to mice exposed to a salt-restricted diet (**Figure 3C**). Cardiovascular comorbidity including increased blood pressure is reported in patients with rheumatoid arthritis (33). To assess the impact of a LS diet compared to a HS diet on the blood pressure, we measured the blood pressure in CIA mice and found significantly reduced blood pressure levels in the LS group compared to the HS group (**Figure 3D**). The measurements of Na^+^ accumulation in the skin can be used to monitor NaCl intake (14). Mice fed with a LS diet showed significant lower concentration of Na^+^ in skin compared to HS diet (**Figure 3E**), consequently indicating a lower salt intake during the experiments whereas concentrations of Na^+^ in plasma did not differ (**Figure 3F**).

To conclude, reducing salt intake exerts beneficial effects on clinical symptoms in CIA, a model that comprises all phases of arthritis development, i.e., the immunization phase driving autoantibody production and the innate effector phase driving joint inflammation.

### Low-Salt Diet Ameliorates Severity of Serum Transfer-Induced Arthritis and Is as Effective as IL-1 Receptor Blockade

**Figure 4:**
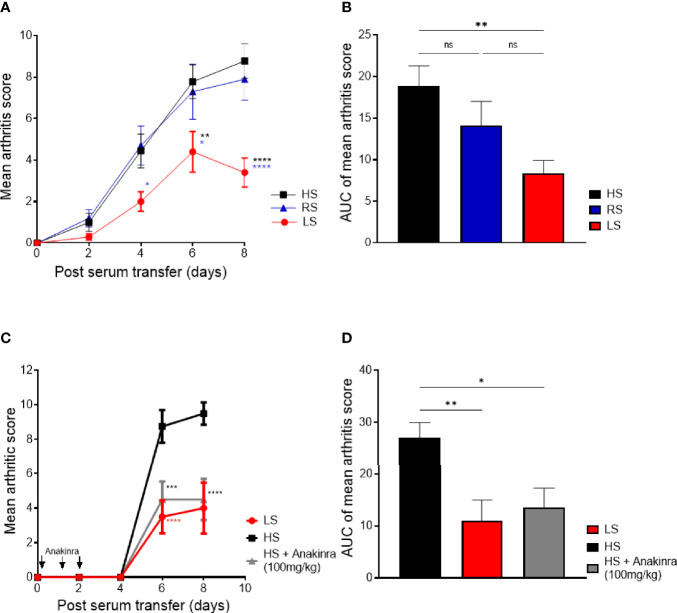
Low-salt diet reduces severity in K/BxN serum transfer-induced arthritis and is as effective as IL-1 receptor blockade. Mice were exposed to low salt (LS), regular salt (RS), and high salt (HS) diets for 2 weeks before serum transfer and continued for further 8 days. **(A)** Mean arthritis score in STIA mice on different diets (LS, *n* = 10; HS, *n* = 10; RS, *n* = 10). *p < 0.05, **p < 0.01,****p < 0.0001; blue asterisk: LS versus RS, black asterisk: LS versus HS. *p*-values were calculated by two-way ANOVA followed by Bonferroni’s multiple comparisons test. **(B)** Area under the curve of mean arthritis score. **p < 0.01; ns = non-significant. *p*-values were calculated by one-way ANOVA followed by Bonferroni’s multiple comparisons test. **(C)** LS diet is as effective as treatment with IL-1 receptor antagonist protein (anakinra) in HS diet mice. One group of mice under HS diet received i.p. injections of 100 mg/kg anakinra simultaneously with arthritis induction as well as on days 1, 2, and 3 (LS, *n* = 4; HS, *n* = 4; HS +Anakinra, *n* = 4). Red asterisk: LS versus HS, gray asterisk: HS/anakinra versus HS. ***p < 0.001, ****p < 0.0001. *p*-values were calculated by two-way ANOVA followed by Bonferroni’s correction. **(D)** Area under the curve of mean arthritis score of STIA mice undergoing LS diet, HS diet, and HS plus anakinra.

Next, we investigated if a low-salt diet also influences arthritis severity in STIA, which mainly resembles the innate effector phase (34). Also in STIA, salt restriction significantly reduced joint swelling compared to HS and RS diets (**Figure 4A**). High NaCl consumption did not aggravate arthritis severity compared to RS diet (**Figure 4A**). The cytokine IL-1 beta, which is predominantly produced by innate immune cells during the effector phase of arthritis, is a key driver of joint inflammation and destruction in RA and, even more, in murine arthritis (1, 29, 35). The IL-1 receptor antagonist–Fc fusion protein anakinra has been approved for RA therapy (36, 37). In STIA, we could show that the LS diet was as effective as IL-1 receptor blocking in HS fed mice (**Figure 4C**), emphasizing the strong beneficial impact of LS diet on the effector phase of murine arthritis. The significant treatment effect of reduced NaCl intake is further demonstrated by the reduced area under the curve (AUC) of the arthritis score (**Figures 4B, D**).

These findings suggest that lowering NaCl intake modulates the myeloid-dependent effector phase resulting in a beneficial effect on arthritis. Of note, also in STIA, excess NaCl intake did not aggravate the disease course compared to regular NaCl intake.

### Low-Salt Diet Rescues Inflammation and Cartilage Destruction Predominantly in Antibody-Mediated Autoimmunity

**Figure 5:**
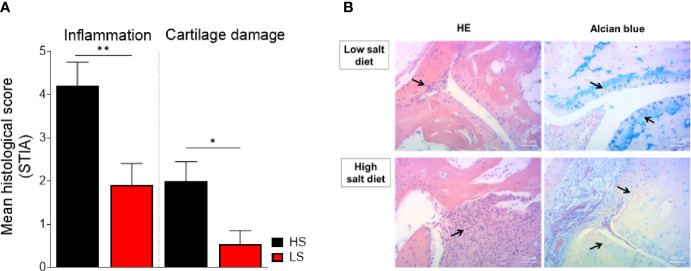
Preventive effect on cell infiltration and cartilage loss by low salt diet in STIA. Paraffin-embedded sections of hind paws were subjected to hematoxylin/eosin (HE) and alcian blue staining to investigate the degree of inflammation and cartilage damage, respectively. Parameters were scored using a three-graded scale and expressed as mean histological score. **(A)** Mean histological scores of inflammation and cartilage loss are shown in the differently treated STIA mice (LS, *n* = 10 mice; HS, *n* = 10 mice). *p < 0.05, **p < 0.01. *p*-values were calculated by unpaired Student’s *t*-test. All data were expressed as ± SEM. **(B)** Representative histopathological images of hind paws stained with HE (left panel) and alcian blue (right panel) of STIA mice on LS diet (upper row) and HS diet (lower row). Arrows indicate pannus formation (HE-stained sections) and cartilage structure (alcian blue-stained sections).

Next, we investigated if the observed LS-induced beneficial clinical effects on arthritis score are reflected in histological evaluation. To that end, histopathological changes were assessed according to a graded scale in hind paws from STIA and CIA mice fed HS and LS diets. In STIA, LS-fed mice showed significantly reduced joint inflammation and cartilage destruction compared to HS-fed mice (**Figure 5A**). In accordance with the clinical observations, we did not observe significant differences regarding inflammation and cartilage loss in HS diet compared to RS diet (data not shown). Representative images of HE and alcian blue-stained joint sections, depicting the degree of joint inflammation and cartilage loss, respectively, of LS diet and HS diet STIA mice, are shown in **Figure 5B**. In CIA we also observed reduced joint inflammation and less cartilage damage upon NaCl restriction; however, histological scores between groups did not reach statistical significance (**Supplementary Figure 2**). In CIA, the beneficial effects of LS diet were more pronounced in the front paws, which were used for cytokine measurements, compared to the hind paws, which were used for histological examination. Therefore, histological examination of front paws instead of hind paws might have resulted in significant differences also in CIA.

Altogether, joint inflammation and the preservation of the cartilage structure can be improved by restricted salt consumption.

As we did not identify relevant differences in clinical or histological parameters between HS and RS diet groups, further analysis was only performed on tissues and organs of LS and HS fed mice.

### Low-Salt Diet Inhibits Anti-CII IgG2a Antibody Production

**Figure 6:**
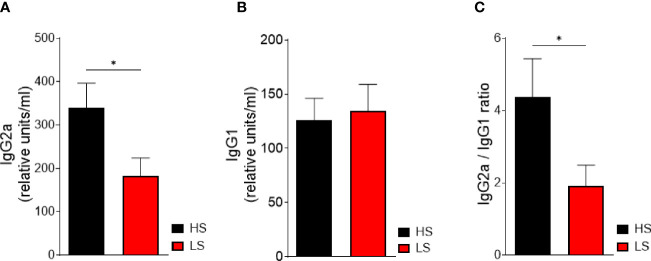
Low-salt diet decreases pathogenic anti-CII IgG2a antibody production and points toward a more Th2-dependent immune response. Serum levels of anti-CII-IgG2a **(A)** and anti-CII-IgG1 **(B)** were analyzed by ELISA in mice undergoing LS and HS diet for 62 days (HS, *n* = 16; LS, *n* = 16). For normalization, a standard was generated containing high titers of anti-CII antibody sera and considered equivalent to 100 arbitrary units/ml. **(C)** The ratio of anti-CII IgG2a to anti-CII IgG1 concentration was determined. *p < 0.05. *p*-values were calculated by Student’s *t*-test. All data were expressed as ± SEM.

Complement-fixing anti-CII IgG2a antibodies are associated with inflammatory cell infiltration and cartilage damage (32, 38). To explore the effect of salt restriction on the adaptive immune response, we determined autoantibody production against CII. Indeed, we observed significantly decreased anti-CII IgG2a serum levels in LS-treated mice compared to HS-treated mice (**Figure 6A**). The serum levels of anti-CII IgG1 did not differ between the LS and HS diets (**Figure 6B**). However, a reduced anti-CII IgG2a/IgG1 ratio pointed toward a more Th2-like response in the LS group (**Figure 6C**).

Taken together, LS diet attenuates arthritis by reducing the humoral response to CII and facilitates a more protective Th2-like response.

### Anti-Inflammatory Features of LS Diet Are Associated With Decreased Numbers of IL-1 Beta- and IL-17A-Expressing Cells in Joints

**Figure 7:**
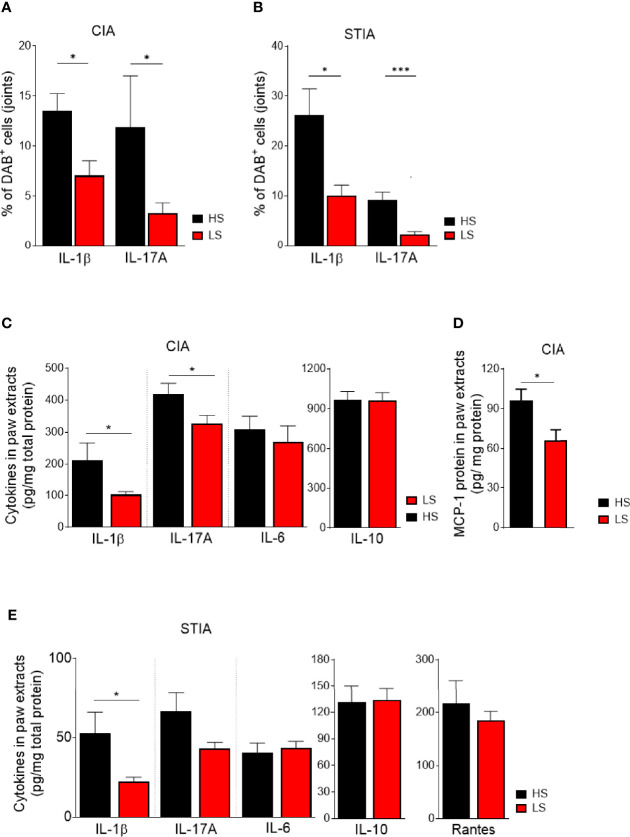
Analysis of cytokine expression in joints. Paraffin-embedded sections of CIA **(A)** and STIA **(B)** hind paws were immunohistochemically stained for IL-1beta, IL-17A, and isotype control. Positive cells were stained in brown (3,3′-diaminobenzidine 4-HCl, DAB). Hematoxylin (blue) was used for counterstaining. Quantification of IL-1beta and IL-17A stained joint sections. Sections of four randomly chosen mice of each group from one experiment were used and three fields per section were analyzed. *p < 0.05, ***p < 0.001. *p*-values were calculated by Student’s *t*-test. **(C)** Front paw extracts were prepared at day 47 post CII immunization and analyzed for IL-1beta, IL-17A, IL-6, and IL-10. **(D)** MCP-1 content (day 20) by ELISA (day 47: LS, *n* = 16; HS, *n* = 14; day 20: LS, *n* = 9; HS, *n* = 10). *p < 0.05. p-values were calculated by Student’s t-test. **(E)** Front paw extracts from STIA mice were isolated at 8 days post serum transfer and analyzed for the respective cytokines by ELISA (HS, *n* = 16; LS, *n* = 16). *p < 0.05. *p*-values were calculated by Student’s *t*-test. All data were expressed as ± SEM.

Pro-inflammatory cytokines such as IL-1 beta and IL-17A contribute to arthritis pathogenesis and trigger joint inflammation (1). To explore the impact of salt restriction on pro-inflammatory cytokine production in the joint, we analyzed IL-1 beta and IL-17A by immunohistochemistry in HS- and LS-fed mice. In both CIA and STIA, assessment of anti-IL-1 beta-stained joint sections showed significantly lower percentages of DAB^+^ cells located within the synovial hyperplasia and pannus of LS compared to HS fed mice (**Figures 7A, B**). Furthermore, the percentage of DAB^+^ cells expressing IL-17A was not only significantly reduced upon LS diet in CIA but also in STIA, although IL-17-producing Th17 cells are unlikely to be involved in the pathogenesis of the STIA model (**Figures 7A, B**). Representative images of IL-1 beta-, IL-17A-, and isotype control-stained paraffin sections of HS and LS hind paws in CIA and STIA are shown in **Supplementary Figure 4A** (CIA) and **Supplementary Figure 4B** (STIA), respectively.

To further corroborate these findings, we assessed concentrations of the pro-inflammatory cytokines, IL-1 beta, IL-17A, and IL-6, as well as the immune-regulatory cytokine IL-10 in tissue extracts of front paws. LS feeding for 62 days (day 47 post CII immunization) significantly decreased IL-1 beta and IL-17A concentrations compared to HS diet, whereas IL-6 and IL-10 concentrations were unaltered (**Figure 7C**). Still, unaltered levels of IL-1 beta, IL-17A, and IL-6 were found in the immunization phase of CIA (day 20 post CII immunization) (**Supplementary Figure 3A**). On the other hand, levels of MCP-1 were significantly reduced upon NaCl restriction at day 20 post CII immunization (**Figure 7D**), whereas MCP-1 levels at day 47 post CII immunization were not significantly reduced upon LS diet (**Supplementary Figure 3B**). Likewise, dietary salt restriction for 23 days significantly lowered IL-1 beta concentrations in the paws of STIA mice, whereas the analysis of IL-17A protein in front paws was numerically reduced but did not reach statistical significance (**Figure 7E**). At the time of analysis, levels of IL-6, IL-10, and RANTES were not affected by salt restriction (**Figure 7E**).

Th17 cells may play a critical role in the pathogenesis of RA and even more in psoriatic arthritis (39–41). The relevance of high salt in Th17 differentiation and production of IL-17 was demonstrated in EAE and in CIA (17, 42). Although arthritis severity was not exacerbated by HS diet compared to RS diet in our CIA studies, IL-17A expression was analyzed in splenic CD4^+^-T cells of CIA mice by flow cytometric analysis. At time of analysis, we did not see significant differences of IL-17A expression in CD4^+^ T cells between the LS, RS, and HS groups (**Supplementary Figure 5A**). In contrast, immunohistochemistry of spleens revealed that LS diet resulted in significantly lower percentages of DAB^+^ cells expressing IL-17A compared to HS diet (**Supplementary Figure 5B**). Furthermore, IL-17A expression was primarily located in the red pulp as well as in the marginal zone of the spleens of CII-immunized mice (**Supplementary Figure 4C**, second row). Unlike in CIA, in STIA, LS feeding resulted in significantly fewer areas of F4/80^+^ macrophages that overlapped with IL-17A^+^ regions (**Supplementary Figure 5C**, **Supplementary Figure 4D** second row). These results suggest that increased IL-17A expression upon HS diet does not predominantly originate from CD4^+^ T cells. In both CIA and STIA, we detected significantly lower numbers of IL-1 beta^+^ cells within the entire red pulp in LS- compared to HS-exposed mice (**Supplementary Figures 4C, D**, first row; **Supplementary Figures 5B, C**).

In summary, these data suggest that in both arthritis models, the beneficial effect by a low-salt diet on arthritis might be a result of a reduced immune cell migration rather than an inhibition of Th17 cell differentiation. Moreover, lower levels of MCP-1 in joints of CIA support these findings and might indicate an “unactivated” status of innate immune cells correlating with reduced migration of leukocytes into affected joints.

### LS Diet Modulates the p38 MAPK/NFAT5 Signaling Axis in Spleen Cells of STIA Mice

A complex network of signaling pathways and transcription factors contributes to the development of arthritis (43). Experiments to analyze mRNA expression levels in spleens of arthritic mice were conducted to get insights into mechanisms that were altered by LS diet compared to HS diet. Results of qRT-PCRs are summarized in **Table 2**. In STIA (8 days post serum transfer), SGK1 and NF-kappaB inhibitor alpha (NF-kappaBIa) mRNA expression did not differ between the groups whereas significantly lower levels of MAPK14 (p38 alpha) and NFAT5 mRNA were detected in the LS group. IL-1 beta mRNA expression was significantly reduced upon LS diet compared to HS diet, whereas IL-17A mRNA levels did not significantly differ between the groups. In spleens of CII-immunized mice (47 days post CII immunization) no differences were found in SGK1, MAPK14, NFAT5, and NF-kappaBIa mRNA expression. Surprisingly, in CIA, relative expression of IL-1 beta and IL-17 mRNA did not differ significantly between LS and HS groups. However, we detected significantly reduced IL-23 mRNA levels under LS diet. As IFN-gamma induces Ig class switch to IgG2a, IFN-gamma mRNA expression was analyzed at day 20 and day 47 post CII immunization. In comparison to HS-diet, NaCl restriction showed a trend toward a reduction of the relative expression of IFN-gamma mRNA at day 20, whereas IFN-gamma mRNA expression was similar in both groups at day 47 (**Table 2****)**.

**Table 2:** Fold change in mRNA expression in spleens.

Mouse model	GENE	Diet		*p*-value
		HS	LS	
	IL-1beta	1.018 ± 0.072	0.667 ± 0.091	0.0079
	IL-17	1.266 ± 0.314	1.709 ± 0.547	0.4930
	MAPK14	1.014 ± 0.061	0.7163 ± 0.158	0.0040
**STIA**	NFAT5	0.981 ± 0.058	0.578 ± 0.120	0.0004
	SGK1	1.023 ± 0.073	0.916 ± 0.162	0.5580
	NF-kappaB-I-alpha	1.186 ± 0.2103	0.9219 ± 0.178	0.3530
	IL-1beta	1.056 ± 0.118	1.324 ± 0.115	0.1240
	IL-17	1.242 ± 0.812	2.092 ± 1.162	0.0855
	IFN-gamma^day47^	1.049 ± 0.110	1.1 ± 0.119	0.8531
	IFN-gamma^d20^	1.051 ± 0.171	0.689 ± 0.090	0.0953
**CIA**	IL-23	1.124 ± 0.1886	0.597 ± 0.093	0.0194
	MAPK14	1.022 ± 0.139	0.760 ± 0.077	0.1201
	NFAT5	1.010 *±* 0.085	0.831 ± 0.125	0.5427
	SGK1	1.018 *±* 0.209	0.908 ± 0.428	0.7495
	NF-kappaB-I-alpha	1.135 ± 0.0552	0.824 ± 0.037	0.6355

STIA and CIA mice fed with high salt (HS) or low salt (LS) diets. Splenocytes were prepared 8 days post serum transfer and 47 days post CII immunization. Fold change expression was calculated by 2^−ΔΔCT^. Gene expression was normalized against HPRT. HS group was used as calibrator (STIA: LS, n = 10; HS, n = 10; CIA: LS, n = 10; HS, n = 9). Statistical significance was defined as p ≤ 0.05. p-values were calculated by Student’s t-test. All data were expressed as ± SEM.

**Figure 8:**
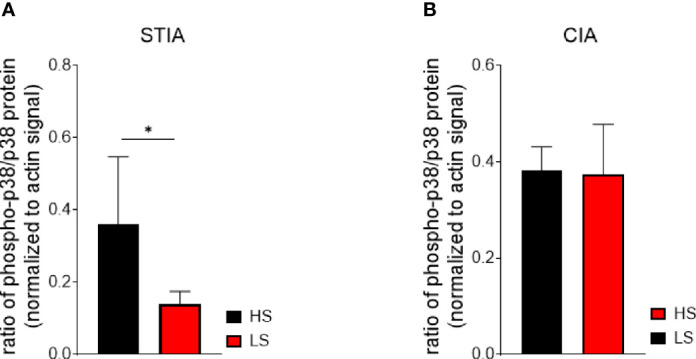
Activation of p38 MAPK is inhibited in the effector phase of arthritis (STIA) by LS diet. Whole splenocyte lysates of STIA **(A)** and CIA **(B)** mice were immunoblotted for phospho-p38 MAPK, p38 MAPK, and beta-actin. Ratios of phospho-p38:p38 were calculated from experimental signals (LS, *n* = 5 HS, *n* = 5). *p < 0.05. *p*-values were calculated by Student’s *t*-test. All data were expressed as ± SEM.

The activation of the p38 MAPKs is caused by environmental stress and cytokines (44–46), thereby regulating many processes such as the production of cytokines and pro-inflammatory mediators as well as migration of leukocytes (47, 48). The relevance of the p38 pathway has been described for arthritis (45, 46, 49, 50). We therefore assessed the ratio of phospho-p38 to p38 total protein in the spleens of STIA and CIA mice by Western blot and detected a significantly lower ratio of phospho-p38 to p38 under LS diet compared to HS diet, indicating an alteration in MAPK signaling (**Figure 8A**). In contrast, no differences between the diets were found in CIA at time point of analysis (**Figure 8B****)**.

Taken together, our data imply that LS diet modulates the p38 MAPK/NFAT5 signaling axis in the effector phase of arthritis, which is accompanied by a reduction of IL-1 beta expression resulting in decreased inflammation. In CIA, innate and adaptive immune mechanisms essentially contribute to arthritis. Moreover, LS diet might not inhibit p38 MAPK/NFAT5 activation. This fact indicates a different underlying mechanism contributing to the observed LS-mediated anti-arthritic effects in CIA.

### Thioglycollate-Elicited Extravasation of Leukocytes Is Reduced Under Sodium Restriction

**Figure 9:**
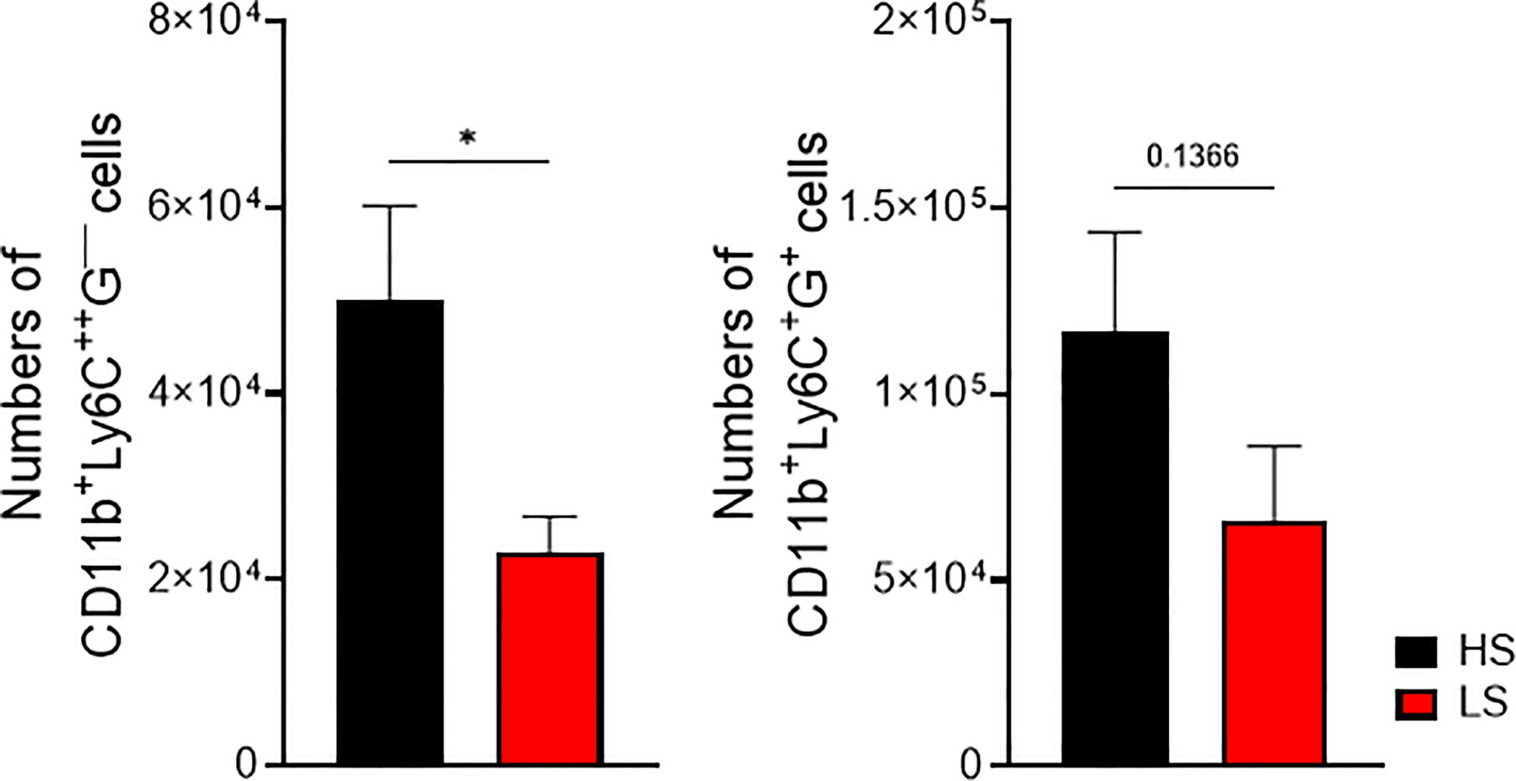
Inhibition of leukocyte extravasation by low-salt diet in thioglycollate-induced peritonitis. Mice were fed with low and high salt diets for 2 weeks before inducing peritonitis. Peritoneal fluid was harvested and analyzed for CD11b^+^Ly6C^++^Ly6G^-^ and CD11b^+^Ly6C^+^Ly6G^+^ infiltrated cells by flow cytometry (LS, *n* = 13 HS, *n* = 12). *p < 0.05. *p*-values were calculated by Student’s *t*-test. All data were expressed as ± SEM.

Migration of leukocytes from the periphery into the affected tissue is a hallmark of inflammation. In order to test whether salt restriction influences the recruitment of leukocytes into the peritoneal cavity, mice were kept under LS and HS diets for 2 weeks before intraperitoneal injection of thioglycollate. Significantly lower numbers of CD11b^+^Ly6C^++^G^─^ cells were recovered upon LS diet whereas numbers of CD11b^+^Ly6C^+^G^+^ cells did not differ significantly between HS- and LS-treated mice (**Figure 9**). However, under LS diet, a trend toward lower numbers of peritoneal CD11b^+^Ly6C^+^G^+^ cells was observed (**Figure 9**).

We suggest that preconditioning of innate immune cells and possibly endothelial cells by salt restriction evokes a reduced recruitment of leukocytes into the inflamed tissue, which might explain the findings of reduced numbers of inflammatory cells in joints of arthritic mice under LS conditions.

## Discussion

Recent, studies provided evidence that excess salt intake is a critical environmental factor contributing to the development of autoimmune diseases such as multiple sclerosis (MS) and inflammatory bowel disease (9–11). Limited studies exist exploring the role of salty food in RA (8, 17). The relevance of salt restriction on clinical features of experimental arthritides has not been explored so far.

Our study demonstrates for the first time that LS diet ameliorates arthritis in two murine arthritis models (**Figures 3**, **4**) when compared to HS and RS diet. Salt restriction reduced joint infiltration of cytokine expressing inflammatory cells whereby lower concentrations of local pro-inflammatory cytokines (IL-1 beta and IL-17) were detected. In antibody-mediated autoimmunity LS diet exerts significant protective effects on cartilage structure. Interestingly, and in contrast to previous reports on EAE (8, 9), our experimental settings did not result in an increase in arthritis severity in mice exposed to HS diet compared to RS diet, neither in the predominantly macrophage-dependent STIA nor in the Th17-dependent CIA.

In EAE, HS diet provoked a more severe disease than a regular diet through increased Th17 differentiation by a salt-dependent induction of SGK-1 (9). Furthermore, enhanced inflammation and cytokine production from intestinal cells were also found in Th17-dependent experimental colitis when salt consumption was increased (10, 11, 51). However, not only lymphoid but also myeloid cells are salt-sensitive and contribute to CNS and intestinal pathogenesis (8, 11). The pathophysiological relevance of NaCl-primed macrophages that mainly adopted a pro-inflammatory M1 phenotype, was highlighted by the fact that EAE worsened upon transferring these macrophages in the absence of activated T cells (8). However, so far only few clinical studies have investigated the impact of salt consumption on inflammatory autoimmune diseases. A clinical study in MS patients suggested that salt consumption exceeding the WHO recommendations (>4.8 g/day) increased clinical and radiological disease activity (52).

In humans, a nested case–control study revealed an increased RA risk among smokers consuming HS diet, which was associated with increased serum concentrations of anti-citrullinated peptide/protein antibodies (15). However, studies exploring the impact of dietary salt on clinical and histological manifestations in experimental arthritides are scarce. Moreover, the studies were based on different experimental settings and mouse strains. Vaartjes et al. reported that the development of arthritis was similar in mice with a moderate salt consumption (1% NaCl in drinking water) to that of regular chow. Examinations in EAE demonstrated that moderate salt consumption did not aggravate clinical manifestations. On the other hand, inconsistent observations were made in the initial phase of collagen-antibody-induced arthritis (CAIA), which is associated with neutrophil and macrophage infiltration into affected joints. Mice exposed to higher salt levels manifested less severe signs of arthritis. However, after LPS stimulation and increased involvement of macrophages, no differences between the groups were detectable (11).

A study that also examined the impact of excess and regular salt intake on CIA was conducted by Jung et al. (17). In their experimental setup (start of feeding 7 days before first CII immunization with 1% NaCl in drinking water plus 4% NaCl chow at the first immunization, followed by a booster immunization), HS diet exacerbated clinical and histological manifestations of arthritis by affecting Th17 differentiation (17). In contrast to these findings, we did not detect differences between the HS, RS, and LS groups when studying the expression of IL-17A in CD4^+^ T helper cells in spleens of CIA mice (**Supplementary Figure 5A**). Possibly these divergent findings are explained by the already relatively high levels of immune cell activation in our RS-treated groups. Also, our data are in line with the findings of Vaartjes et al. (11). However, it is not clear if the discrepancy between our immunization protocol and experimental settings (HS diet: feeding 1% NaCl in drinking water plus 4% NaCl chow starting 14 days before single CII immunization or serum transfer until sacrifice day) explains the differences in Th17 differentiation. Of note, based on our results in experimental arthritis, we did not include the RS groups in further investigations.

CII immunization in CIA activates innate and adaptive immune responses, including Th cells, whereas pathogenic CII-specific autoantibodies drive disease progression together with innate immune cells (32, 38). Similar to STIA, which is a T- and B-cell-independent model, CII immunization eventually leads to immune complex (IC) formation followed by complement activation attracting neutrophils, monocytes/macrophages, and mast cells into the joint space (18). Consequently, joint inflammation occurs through the release of inflammatory cytokines such as IL-1 beta and IL-17 (35, 53). Accordingly, the adaptive humoral immune response in CIA was reduced and we detected significantly lower levels of anti-CII IgG2a antibodies with LS diet compared to HS diet and a reduced IgG2a:IgG1 ratio of anti-CII auto-antibodies in the LS groups (**Figure 6**). This fact may indicate that LS diet favors a shift from a Th1- toward a Th2-dominated response. Recently, Dahdah et al. identified GC formation and anti-collagen antibody production as key pathogenic functions of B cells in CIA by the use of genetically modified animals (54). Furthermore, inducing arthritis in GC-deficient mice by injection of pathogenic antibodies resulted in decreased arthritis. Therefore, the authors suggested that GC B cells might have additional functions such as antigen presentation, which are relevant to disease development (54). The crosstalk of T follicular helper (Tfh) cells and germinal center (GC) B cells triggers antibody-producing plasma cells and memory B cells (55). Salt restriction might also influence GC B functionality leading to reduced production of pathogenic anti-CII antibodies. The regulation of Tfh and GC B cell function in CIA was not investigated in this study but is worthwhile to be addressed in the future.

The use of two different experimental arthritis models enabled us to distinguish the effects of dietary salt intake on the immunization phase from the effector phase driven by cells of the innate immune system like in STIA. In the effector phase of RA and murine arthritis, the pro-inflammatory cytokine IL-1 beta is expressed by monocytes/macrophages and contributes to arthritis pathogenesis for instance due to chondrocyte activation followed by cartilage damage (56–58). The recombinant human IL-1 receptor antagonist-Fc fusion protein anakinra is approved for RA treatment and suppresses the inflammatory response (1). Our data demonstrate that salt restriction reduced the severity of STIA as efficiently as IL-1R blockade in HS-fed mice (**Figures 4C, D**), suggesting that salty diets activate cells of the monocyte/macrophage lineage. *Vice versa*, sodium chloride restriction possibly inhibits the activation status predominantly of innate immune cells.

Histological analysis of the hind joints revealed reduced joint infiltration of IL-1 beta and IL-17A expressing inflammatory cells in both models (**Figures 7A, B**) whereby significantly lower concentrations of local IL-1 beta (**Figures 7C, E**) were detected in the LS diet groups. Accordingly, we found that salt restriction significantly reduced IL-17A production in the joints of CIA mice and markedly lowered IL-17A production in STIA (**Figures 7C, E**). In line with that, IL17R-s**ignaling** was shown to play an important role as amplifier of the effector phase of inflammatory arthritis (59). In STIA, we observed F4/80^+^ macrophages, which overlapped in immunohistology with the IL-17A^+^ region (**Supplementary Figure 3D**). Moreover, IL-17A expression was mainly located in the red pulp as well as in the marginal zone of the spleen, compartments in which macrophages were usually predominant. The fact that IL-17A rather originates from innate immune cells than CD4^+^ T cells, at least in our experimental setting, is supported by previous studies of Scrivo and colleagues, who reported no significant reduction in the frequencies of peripheral Th17 cells in RA patients upon LS diet (16).

In *in vitro* assays, we found decreased TNF-alpha and IL-6 expression under low salt conditions in LPS-stimulated murine monocytes whereas equi-osmolalic solutions of mannitol did not increase the cytokines compared to regular (125 mM NaCl; 299 mOsm/kg) and high salt conditions (165 NaCl; 387 mOsm/kg, **Figure 1**). Although *in vitro* culture of cells at low salt concentrations might induce different mechanisms leading to anti-inflammatory features, the results further support our *in vivo* data suggesting that preconditioning of predominantly myeloid cells at low salt concentrations inhibits their activation.

An inflammatory response is characterized by transmigration of leukocytes through the cytokine-activated endothelium into the affected tissue. We observed decreased accumulation of CD11b^+^Ly6C^++^Ly6G^-^ cells in the peritoneal cavity upon LS diet compared to HS diet in the model of thioglycollate-induced peritonitis (**Figure 9**). The chemokine MCP-1 is expressed predominantly by endothelial cells, fibroblasts, and monocyte-macrophages and plays a critical role in orchestrating the influx of leukocytes into sites of inflammation. In RA patients, high expression of MCP-1 is found within the joints (60–62). An animal study (CIA) of Ogata et al. showed that MCP-1 is involved in joint destruction by recruitment of monocytes (63). During the immunization phase, we detected decreased MCP-1 protein in the joints of CIA mice fed with LS diet (**Figure 7D**). Extravasation is not only regulated by chemoattractant proteins, but also by adhesion molecules such as E-selectin mediating the interaction between leukocytes and endothelium (64). Therefore, we addressed the question if low salt conditions can modulate E-selectin expression on endothelial cells *in vitro*. Our analysis revealed that cytokine-induced E-selectin expression on endothelial cells is upregulated in a salt-dependent manner and can be inhibited under low salt conditions suggesting that the surrounding hypotonic milieu might modulate the endothelial status toward a decreased leukocyte extravasation. Recently, Lin et al. showed that E-selectin expression is transcriptionally regulated *via* the NF-kappaB/JNK pathways (31). We demonstrated that salt restriction did not inhibit IL-1 beta-induced NF-kappaB activation in endothelial cells *in vitro*, suggesting a NF-kappaB-independent but a possibly JNK-dependent downregulation of E-selectin expression (**Figures 2B, C**). Also here, we did not see differences between regular and high-salt conditions. Previously it was shown that elevation of extracellular NaCl upregulates pro-inflammatory mediators in endothelial cells in culture and increases their adhesive properties. Excess salt concentrations promote PBMC adhesion and transmigration in human umbilical vein endothelial cells (HUVECs). Dmitrieva and colleagues demonstrated that water restriction *in vivo* increased serum sodium by about 5 mmol/L and upregulated VCAM-1, E-selectin, and MCP-1 mRNA in several tissues (65). In contrast to these findings, we found equal plasma sodium ion concentrations in LS and HS mice, even after CII immunization, whereas sodium ion concentrations were markedly increased in the skin of HS mice (**Figures 3E, F**). Therefore, we cannot clearly distinguish if amelioration of arthritis under conditions of salt restriction *in vivo* is due to direct effects on endothelial cells or rather due to a decreased cytokine expression by innate immune cells residing in tissues accumulating sodium ions.

To evaluate possible mechanisms that might contribute to beneficial effects of LS diet on murine arthritis, we performed mRNA gene expression analysis. A previous study showed that hypertonic solutions induce Th17 polarization by activating the p38/MAPK pathway involving TonEBP/NFAT5 and SGK1 (9). In STIA, low-salt diet significantly decreased MAPK14 mRNA expression accompanied by significantly lower mRNA levels of NFAT5, which was not observed in CIA. In both models, LS diets did not alter SGK1 and NF-kappaB-inhibitor alpha mRNA expression (**Table 2**). Moreover, in the myeloid cell-dependent STIA model, activation of p38 MAPK was reduced by LS (**Figure 8A**), which was not seen in CIA (**Figure 8B**). To support the relevance of p38 MAPK signaling under high salt conditions, we performed *in vitro* experiments demonstrating that the presence of the p38 inhibitor SB203580 did not further decrease TNF-alpha and IL-6 production in LPS-stimulated monocytes pre-cultured in LS medium in contrast to RS and HS conditions (data not shown). These data suggest that *in vitro* salt restriction induces an inactive state in monocytes. Analysis of IL-17A gene expression in spleens of CIA and STIA mice showed no differences between HS and LS diets. However, LS diet significantly suppressed IL-1 beta gene expression in spleens of STIA whereas similar levels of IL-1 beta gene expression was observed in spleens of CIA with LS and HS diet. Moreover, qRT-PCR analysis revealed that in the immunization phase of CIA, gene expression of IFN-gamma was reduced. Furthermore, LS diet significantly decreases IL-23. These findings suggest that low-salt diet may inhibit isotype switching and monocyte/macrophage activation, which might contribute to the observed beneficial effects on clinical arthritis.

Our study has several limitations. First, we did not explore in detail the mechanisms leading to reduced IL-1 beta expression under salt deprivation or the effect of IL-1 receptor blockade in LS diet. Second, we did not investigate further the cellular source of IL-17A. All of the above would have been beyond the scope of our study.

To summarize, we showed for the first time that dietary salt restriction has clearly beneficial effects on clinical and histopathological features in murine arthritis, most likely through reduced cell accumulation in the affected joints and lower levels of local IL-1 beta and IL-17 protein. LS diet was proven to be as efficient as IL-1R blockade in reducing disease severity in STIA. In spite of several investigations of signaling pathways shown here, the underlying molecular mechanisms of salt restriction on the complex network of cell types and signaling pathways contributing to arthritis pathogenesis needs to be further explored. Prospective clinical trials are required to investigate the effects of NaCl restriction as a potential supportive therapeutic intervention in human immune-mediated arthritides such as RA and psoriatic arthritis.

## Data Availability Statement

The raw data supporting the conclusions of this article will be made available by the authors, without undue reservation.

## Ethics Statement

The animal study was reviewed and approved by Regierungspräsidium Freiburg Referat 35 Veterinärwesen, Lebensmittelüberwachung Bertoldstr. 43 79098 Freiburg, Germany.

## Author Contributions

Conceived and designed the experiments: BS, JT, and RV. Performed the experiments: BS, RR, CH, SP. Analyzed the data: BS, CH, and MS. Contributed reagents/materials/analysis tools: FN. Wrote the manuscript: BS, NC, and RV. All authors contributed to the article and approved the submitted version.

## Funding

This work was supported by the German Research Foundation (DFG): TRR 130, project 12 to RV; TRR 130 to FN and FOR2886 to FN; SFB 1160, project 12 to RV; and A. Triantafyllopoulou and project Z1 to MS; European Community-funded (ERDF) project INTERREG V “RARENET”; and by the Erika Bürgy Fundation (Stiftung für die Region – Sparkasse Pforzheim Calw Treuhandstiftung Erika Bürgy Stiftung).

## Conflict of Interest

The authors declare that the research was conducted in the absence of any commercial or financial relationships that could be construed as a potential conflict of interest.

## Publisher’s Note

All claims expressed in this article are solely those of the authors and do not necessarily represent those of their affiliated organizations, or those of the publisher, the editors and the reviewers. Any product that may be evaluated in this article, or claim that may be made by its manufacturer, is not guaranteed or endorsed by the publisher.
