# Supplementary material for: Low-Salt Diet Attenuates B-Cell- and Myeloid-Cell-Driven Experimental Arthritides by Affecting Innate as Well as Adaptive Immune Mechanisms
Source: Front Immunol. 2021 Dec 3;12:765741. doi: 10.3389/fimmu.2021.765741 (PMC8678127; doi:10.3389/fimmu.2021.765741)
Supplement: Supplementary file 1 [file DataSheet_1.docx]

Supplementary Material

**SUPPLEMENTARY METHODS**

**Measurement of blood pressure and Na^+^ concentration in skin and plasma**

In CIA, blood pressure as well as skin and plasma Na^+^ concentrations were analyzed at sacrifice day as described by Machnik et al. (1).

**Flow cytometric analysis for CD4^+^IL-17A^+^ T cells**

Mouse splenocytes were cultured in RPMI 1640 (Life Technologies GmbH, Darmstadt, Germany) supplemented with 2 mM glutamine, 10% heat-inactivated fetal bovine serum, 100 IU/mL penicillin/100 μg/mL streptomycin and 50 µM ß-mercaptoethanol (all reagents supplied by Life Technologies GmbH). For intracellular IL-17A staining, 1x10^6^ splenocytes of CIA mice were fixed and permeabilized using Fix/Perm solutions (BD, Heidelberg, Germany) according to the manufacturer’s instructions. Afterwards, cells were stained using CD4- PerCP-Cy5.5 (eBiosience #42-0042-80) and IL-17A-PE (Biolegend #506903). Labeled cells were washed twice in FACS buffer (PBS/2% FCS) and then analyzed using a Coulter Gallios flow cytometer (BeckmanCoulter, Krefeld, Germany). Absolute numbers of CD4^+^IL-17A^+^ T cells were calculated.

**Western blot analysis**

Whole cell lysates of mouse splenocytes were separated by 12% SDS-PAGE. After electrophoresis, the proteins were transferred to PVDF membrane at 70 mA V for 60 min using a Biometra semi-dry transfer cell (Biometra GmbH, Göttingen, Germany). The membranes were incubated in blocking buffer (TBST buffer with 5% BSA) for 1 h at room temperature. Afterwards, the membranes were incubated overnight at 4°C with a rabbit anti- p38 MAPK antibody ([1: 1000], # 9212, Cell Signaling Technology Europe B.V., Frankfurt am Main, Germany) and a rabbit anti-phospho-p38 MAPK antibody ([1:1000], # 4511, Cell Signaling) respectively. Signals were normalized to beta-actin using a mouse anti-actin antibody ([1: 2000], # A1978; Sigma, Taufkirchen, Germany). After washing the membranes five times in TBST the respective IRdye-labelled secondary antibodies (goat anti-mouse IgG(H+L) IR680LT [1:5000] and goat anti-rabbit IgG(H+L) IR800CW [1:5000]; Li-Core Biosciences, Bad Homburg, Germany) were added for 1 h at room temperature. After another five washing steps, the blots were analyzed by an Odyssey Infrared Imaging System (Li-Core Biosciences, Bad Homburg, Germany). Normalized experimental signals were used to represent the ratio of phospho-p38/total p38 (lane normalization factor = observed signal of housekeeping protein for each lane/highest observed signal of housekeeping protein; normalized experimental signal = observed experimental signal/lane normalization factor).

**RNA extraction and quantitative real-time PCR**

Total RNA was purified using the Extrazol Reagent (Blirt, Gdańsk, Poland). 1µg of total RNA was reversely transcribed using the SuperScript® VILO™ cDNA Synthesis Kit according to the manufacturers’ instructions (Life technologies, Frankfurt, Germany) in a total volume of 20 µl. Quantitative real-time PCR (qRT-PCR) was performed using the StepOnePlus Real-Time PCR System (PE Applied Biosystems) to analyze the mRNA gene expression. The following TaqMan probes (Thermo Fisher Scientific) were used: HPRT (Mm00446968_m1), IL-1 beta (Mm00434228_m1), IL-17A, (Mm00439618_m1), IFN-gamma (Mm00477798_m1), IL-23 (Mm01168134_m1), NFAT5 (Mm00467257_m1), SGK1 (Mm00441380_m1), MAPK14 (Mm00442499_m1) and NFkIBa (Mm00477798_m1). Relative changes in gene expression were analyzed by the [2^(-ΔΔCT)]. CT values were normalized using HPRT and the HS diet group was used as calibrator. Data were presented as x-fold change to HS group. PCRs were set-up in triplicates.

**Electrophoretic mobility shift assay (EMSA)**

*mlENDs* were treated in the respective media and stimulated with IL-1 beta (40 ng/ml) for 1 hour as described in the main text of the manuscript. Nuclear proteins of *mlEND* cells were isolated as previously described (2). IRDye700-infrared–labeled double-stranded oligonucleotides containing an NF-kappaB–binding site (5′-AGT TGA GGG GAC TTT CCC AGG C-3′, 3′-TCA ACT CCC CTG AAA GGG TCC G-5′) were used for EMSA analyses and visualized using an Odyssey Infrared Imaging System. Octamer-binding protein 1 (Oct-1) transcription factor was used as loading control (IRDye800-infrared double-stranded oligonucleotides containing an Oct-1 –binding site; 5′ -AGA GAT TGC CTG ACG TCA GAG AGC TAG- 3′, 3′ -TCT CTA ACG GAC TGC AGT CTC TCG ATC- 5′).

**Supplementary FigureS**

**Supplementary Figure 1**

**
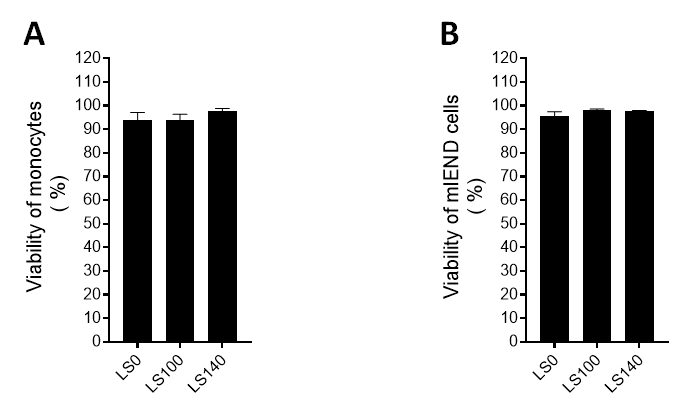
**

**Supplementary Figure 1:** Viability of monocytes and *mlENDs* cultured in medium with different concentrations of NaCl. **(A)** A Trypan blue exclusion assay was performed after LPS stimulation to analyze numbers of viable and dead monocytes cultured in LS0, LS100 and LS140 media. **(B)**  IL-1 beta stimulated *mlENDs* cultured in LS0, LS100 and LS140 media were stained with propidium iodide (PI) and analyzed by flow cytometry. Data of three experiments are shown and expressed as percentage

(%) of viable cells. P values were calculated by one-way ANOVA followed by Bonferroni’s multiple comparisons test. All data were expressed as ± SEM.

**Supplementary Figure 2**


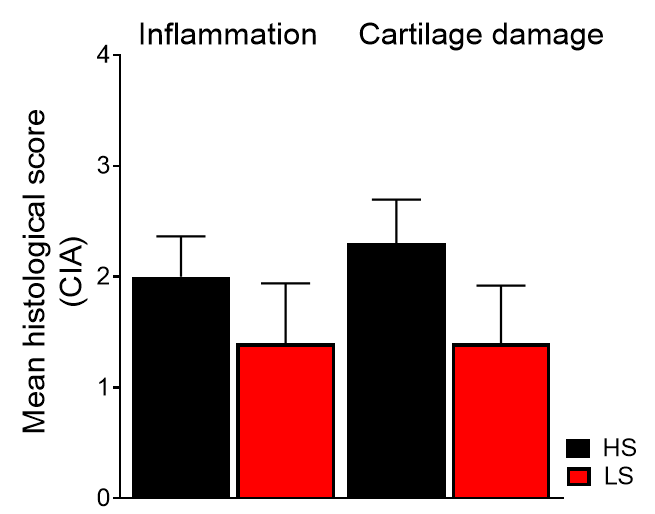


**Supplementary Figure 2:** Assessment of inflammatory cell infiltration and cartilage loss of HE- and Alcian blue-stained sections of CIA hind paws. Mean histological score of inflammation and cartilage loss is shown in differently treated CIA mice (LS, n=19; HS, n=19). *P* values were calculated by Student's t-test. All data were expressed as **±** SEM.

**Supplementary Figure 3**

**
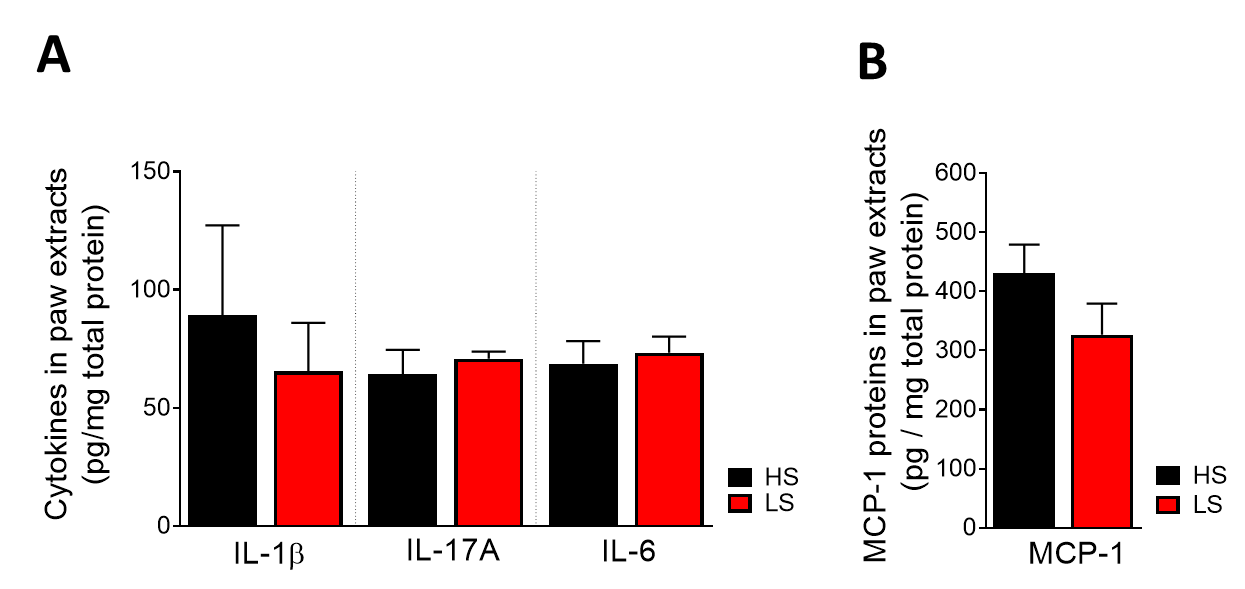
**

**Supplementary Figure 3:** Analyzing of pro-inflammatory cytokines and mediators at day 20 post CII-immunization. **(A)** No alterations of IL-1 beta, IL-17A, and IL-6 levels were observed in joint tissue extracts of CIA between low salt and high salt fed mice on day 20. **(B)** MCP-1 levels on day 47 (day 20: LS, n=9; HS, n=10; day 47: LS, n=16; HS, n=14). *P* values were calculated by Student's t-test. All data were expressed as **±** SEM.

**Supplementary Figure 4**

**
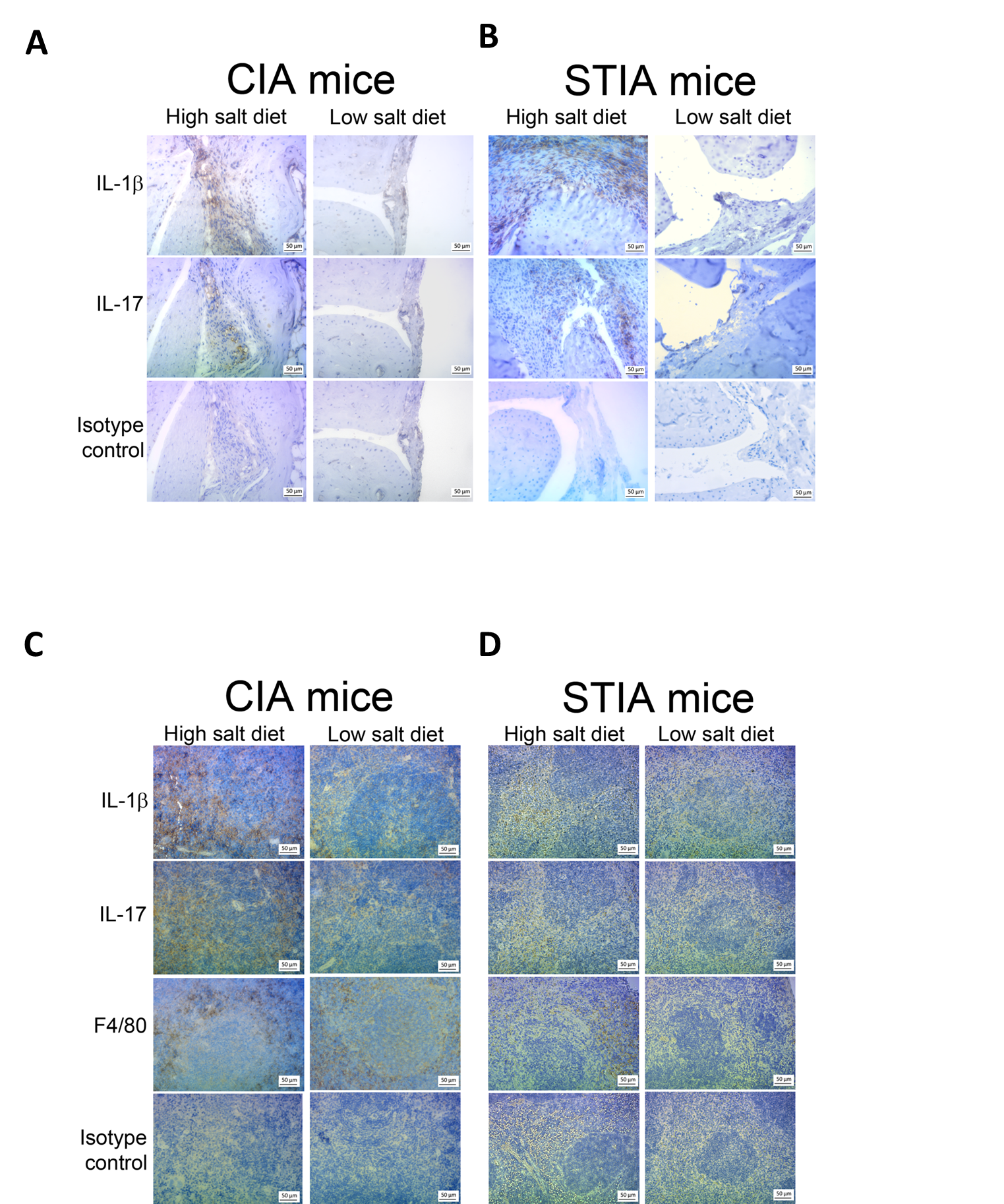
**

**Supplementary Figure 4:** Representative images of paraformaldehyde-fixed paraffin-embedded and immunohistochemically (IHC) stained joint and spleen sections of CIA and STIA mice under LS and HS diets. At the end of the experiments paraffin-embedded sections of hind paws and spleens were prepared. **(A)** Representative images of joint sections of CIA and **(B)** STIA mice under high salt diet (left panels) and low salt diet (right panels) are shown. Cross-sections were immunohistochemically stained for IL-1 beta, IL-17A and isotype control. IL-1 beta, IL-17A^+^ cells were stained in brown color (3,3′-diaminobenzidine 4-HCl, DAB). Hematoxylin (blue) was used for counterstaining. **(C)** Representative images of spleen sections stained for IL-1 beta, IL-17A^+^, F4/80 and isotype control of CIA and **(D)** STIA mice under high salt diet (left panels) or low salt diet (right panels) are shown.

**Supplementary Figure 5**

**
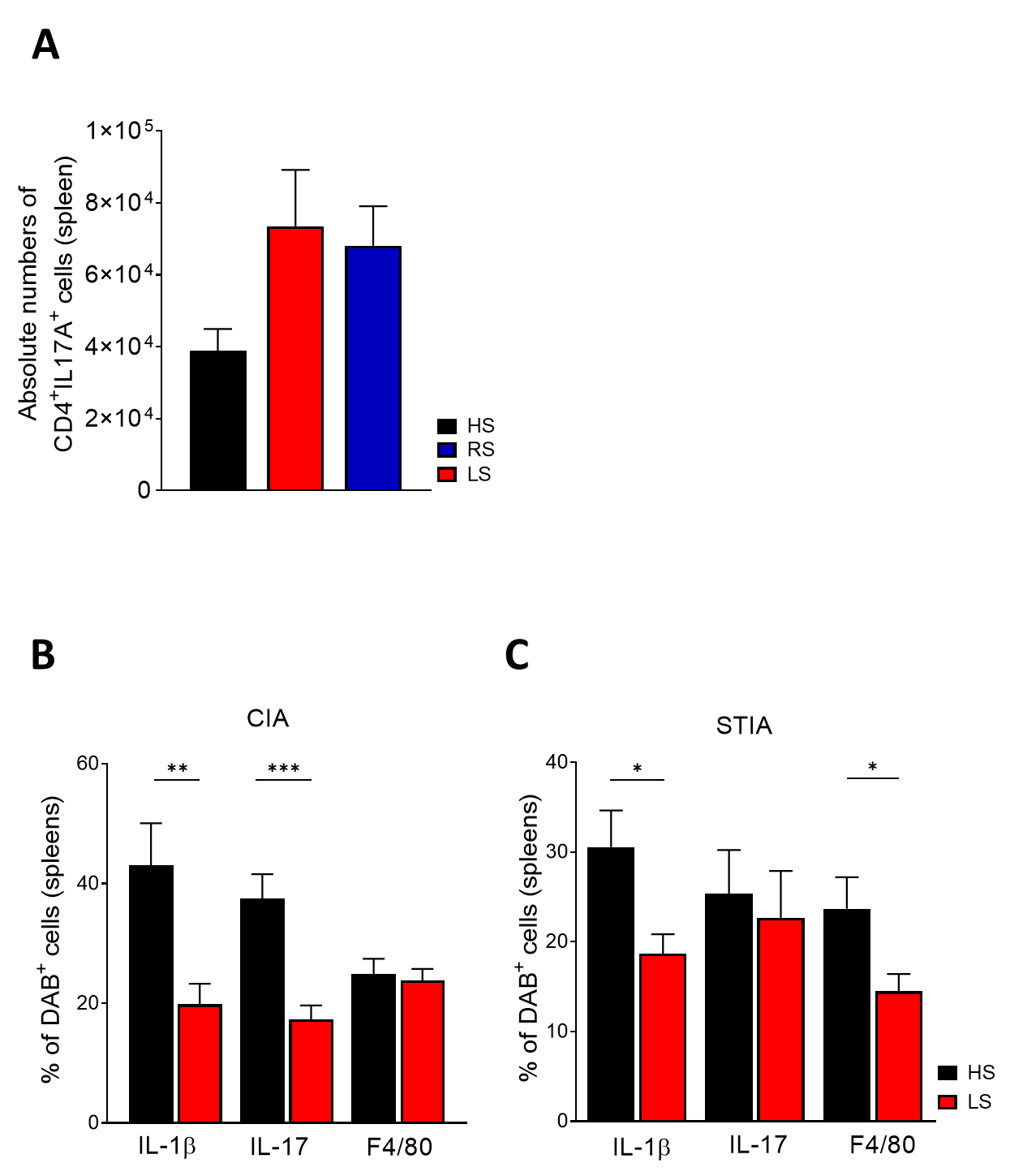
**

**Supplementary Figure 5:** Quantification of IL-1 beta, IL-17A and F4/80 stained spleen sections and FACS analysis of splenic CD4^+^IL-17A^+^ T cells. **(A)** Splenocytes were harvested at day 47 post CII-immunization from CIA mice exposed to HS , RS and LS diets. CD4^+^IL-17A^+^ T cells were analyzed by flow cytometry and absolute numbers were calculated (LS, n=5; RS, n= 5; HS, n=5). *P* values were calculated by one-way ANOVA followed by Bonferroni’s multiple comparisons test. Spleen sections of 4 randomly chosen CIA (**B**) and STIA (**C**) mice of each group from one experiment were used and 3 fields per section were analyzed. Percentages of DAB^+^ stained cells for IL-1 beta, IL-17A^+^ and F4/80 is shown. *= *P* < 0.05, **= *P* < 0.01, ***= *P* < 0.001. P values were calculated by Student's t-test. All data were expressed as **±** SEM.

**Supplementary referenceS**

1. Machnik, A., Dahlmann, A., Kopp, C., Goss, J., Wagner, H., Van Rooijen, N. et al. (2010). Mononuclear phagocyte system depletion blocks interstitial tonicity-responsive enhancer binding protein/vascular endothelial growth factor C expression and induces salt-sensitive hypertension in rats. *Hypertension* 55**,** 755-761.

2. Ghosh, S., and Baltimore, D. (1990). Activation in vitro of NF-kappa B by phosphorylation of its inhibitor I kappa B. *Nature* 344**,** 678-682.
